# Lowbush blueberry fruit yield and growth response to inorganic and organic N-fertilization when competing with two common weed species

**DOI:** 10.1371/journal.pone.0226619

**Published:** 2019-12-26

**Authors:** Charles Marty, Josée-Anne Lévesque, Robert L. Bradley, Jean Lafond, Maxime C. Paré

**Affiliations:** 1 Laboratoire d’écologie Végétale et Animale, Département des Sciences Fondamentales, Université du Québec à Chicoutimi, Saguenay, QC, Canada; 2 Département de Biologie, Université de Sherbrooke, Sherbrooke, QC, Canada; 3 Soils and Crops Research and Development Centre, Agriculture and Agri-Food Canada, Normandin, QC, Canada; Universidade de Santiago de Compostela, SPAIN

## Abstract

Inorganic N fertilizers are commonly used in commercial blueberry fields; however, this form of N can favor increased weed species’ growth, which can ultimately reduce the benefits of fertilization. We hypothesized that chipped ramial wood (CRW) compost is an effective alternative organic fertilizer for blueberry plants when weeds are present, as ericaceous shrub species are generally more efficient in utilizing organic N than herbaceous weed species. In this study, we measured the growth, fruit yield, and foliar N response of lowbush blueberry (*Vaccinium angustifolium* Aiton) to an application of 45 kg N ha^-1^ in the form of organic (CRW) or inorganic N (ammonium sulfate) in two areas of a commercial field colonized by either poverty oat grass (*Danthonia spicata* (L.) Beauv.) or sweet fern (*Comptonia peregrina* (L.) Coult.). We also assessed the impact of the fertilization treatments on litter decomposition rates. Contrary to our hypothesis, we found no significant increase in blueberry fruit yield or growth using CRW. By contrast, inorganic N-fertilization increased fruit yield by 70%. The effect was higher in the area colonized by *D*. *spicata* (+83%) than by *C*. *peregrina* (+45%). Blueberry fruit yield was on average twice higher in the area of the field having *D*. *spicata* than *C*. *peregrina*, suggesting a stronger competition with the latter. However, the increase in *D*. *spicata* density from 0–1 to >25 plants m^-2^ reduced fruit production by three-fold and strongly impacted vegetative growth in both fertilized and unfertilized plots. The impact of increased *C*. *peregrina* density was comparatively much lower, especially on vegetative growth, which was much higher in the area having *C*. *peregrina*. These patterns are likely due to a lower competition for N uptake with *C*. *peregrina* as this species can derive N from the atmosphere. Interestingly, the higher fruit yield in the area colonized by *D*. *spicata* occurred even in plots where the weeds were nearly absent (density of 0–1 plant m^-2^), revealing the influence of unidentified variables on blueberry fruit yield. We hypothesized that this difference resulted from over-optimal foliar N concentrations in the area colonized by *C*. *peregrina* as suggested by the significantly higher foliar N concentrations and by the negative correlation between foliar N concentrations and fruit yields in this area. The possibility of an influence of *C*. *peregrina* on flowering and pollination success, as well as of unidentified local site conditions is discussed. The tested N-fertilization treatments did not affect foliar N concentrations or litter decomposition rates. Overall, our results show that ammonium sulfate is very effective at increasing fruit yields but that both fruit yields and the efficiency of the N-fertilization treatment are decreased by increased *D*. *spicata* density, especially above 25 plants m^-2^. Although CRW did not significantly enhance fruit yields in the short term, this fertilizer may have a long-term beneficial effect.

## Introduction

Wild lowbush blueberry (*Vaccinium angustifolium* Aiton) is a perennial ericaceous shrub species that naturally forms dense and extensive colonies in the boreal forests of eastern North America. Following a major forest disturbance, such as wildfire or clearcutting, this shrub will spread and dominate the vegetative cover due to its extensive rhizomatic network [1]. Lowbush blueberry is grown for its fruits and represents an economically important crop in northeastern North America, especially in Quebec, Maine, and the Canadian Atlantic provinces. Quebec’s commercial production expands over more than 35,000 ha and represented about the third of the Canadian production over the period 2010–2014 (20,373 t yr^-1^) [2].

Inorganic N fertilizers, mainly ammoniacal forms (N-NH_4_^+^), are used in commercial blueberry fields to stimulate vegetative growth and fruit yield [3]. This fertilizer is usually applied in the early spring of the vegetative year [4], and its application is often accompanied by weed control treatments [5]. The presence of weeds tends to diminish yields and the efficacy of inorganic N-fertilization [6] because some weed species are more efficient than blueberries in the taking up of inorganic N [7]. On the other hand, boreal ericaceous shrubs are more efficient in taking up organic N-forms from humus than other boreal forest plants [8,9] due to their association with ericoid mycorrhizal fungi [10,11]. Therefore, we hypothesized that lowbush blueberry is more competitive than companion weed species when fertilized with organic substrates. If true, then the use of organic fertilizers may provide an option for weed management in commercial lowbush blueberry production [12]. This needs to be assessed, however, because blueberry fertilization studies using different organic sources, e.g., peat moss, pine bark, sawdust, hay, manure, paper, leaf litter, plant residues, and compost, have produced inconsistent results. For instance, two studies reported an increase in blueberry yields and foliar nutrient concentrations after fertilizing with paper mill sludge [13,14]. In contrast, Warman [15] found that organic manures had no significant effect on fruit yields or leaf N concentrations, whereas Warman et al. [16] showed that municipal wastes increased foliar nutrients but did not improve yields. It is thus possible that ericoid mycorrhizae associated with lowbush blueberry roots have a greater affinity for organic nutrients derived from forest woody debris, e.g., paper mill sludge, than from other organic sources—manures and municipal wastes. There is a need to evaluate the fertilization potential of other forest-based composts.

Chipped ramial wood (CRW) is an organic material derived from the small branches, bark, and leaves of tree saplings. This woody debris contains nutrients, sugars, celluloses, proteins, and lignin, all of which contribute to the formation of a fertile compost [17]. Composted CRW can improve soil structure, water-holding capacity, and soil biological activity, features that are essential for maintaining healthy crops [18,19]. Several studies have shown the short, medium, and long-term efficacy of CRW compost on agricultural production on different types of soil and in various climates [18]; to our knowledge, however, this type of fertilizer has never been tested in lowbush blueberry fields. Applying CRW compost to lowbush blueberry crops may not only be a means of increasing growth and fruit production but may also alleviate the need for chemical weed control. We thus conducted a study in a commercial lowbush blueberry field in Quebec, where we compared the growth and yield of lowbush blueberry when fertilized with ammonium sulfate or CRW compost in competition with two common weed species, namely poverty oat grass (*Danthonia spicata* (L.) Beauv.; thereafter *D*. *spicata*) and sweet fern (*Comptonia peregrina* (L.) Coult.; thereafter *C*. *peregrina*). These two species are characterized by distinct functional traits which drive the direction and the magnitude of interactions with lowbush blueberry [7]. *D*. *spicata* is a perennial grass (Poaceae) that is native to North America, commonly found in the pastures of southern Quebec and the Maritime provinces where it invades dry-soil and low-fertility agricultural fields due to its numerous, shallow and fibrous roots [20]. In contrast, *C*. *peregrina* is a N_2_-fixing woody shrub of the Myricaceae family that grows 0.3–1.5 m in height which spreads mainly by rhizomes and forms thickets in sunny or partially shady locations. This plant also develops cluster roots, allowing this species to colonize sterile soils, such as abandoned fields and pine barrens, and to grow well on nutrient-depleted—especially P-limited—habitats [21]. This species represents one of the most serious weed problems in the commercial lowbush blueberry fields of eastern Canada [22]. In a previous study, we showed that *D*. *spicata* is a strong competitor for inorganic N fertilizer acquisition due to its shallow and dense root system, whereas *C*. *peregrina* has a beneficial impact on blueberry growth due to its N_2_-fixation ability [7]. However, the latter may be a stronger competitor when N-fertilization occurs as organic N due to its cluster roots. Therefore, we hypothesized that the positive response of lowbush blueberry growth and fruit yield to CRW addition would be stronger in competition with *D*. *spicata* than with *C*. *peregrina*. Conversely, we hypothesized that the presence of *D*. *spicata* would strongly reduce fruit yields in plots fertilized with inorganic N, whereas *C*. *peregrina* would have a minor effect due to its ability to obtain part of its N from the atmosphere.

## Materials and methods

### Study site

The study was conducted in a commercial lowbush blueberry field owned by the St-Eugene Coop (i.e., producers' association) which granted us permission to conduct this research work. This commercial field is located near Saint-Eugène d’Argentenay in the Saguenay–Lac-Saint-Jean region, Quebec, Canada (48°59′N, 72°18′W), which contains 82% of Quebec’s commercial lowbush blueberry surface area [2]. A study was previously conducted on this field to assess the ability of the three aforementioned species to acquire ^15^N from an inorganic fertilizer [7]. Soils in the lowbush blueberry stands are podzols characterized by fine sands that originate from fluvioglacial deposits. The soils are very acidic and have a low water retention capacity; they are thus non-fertile [23]. The region is characterized by a cold and humid climate having a mean annual air temperature of 0.8°C and annual precipitation of ~948 mm. Mean precipitation and air temperature during the growing season (May–August) are 346 mm and 14°C, respectively. The studied lowbush blueberry field was established in 2005 and has an average stem density of ca. 500 m^-2^. The blueberry crop is currently cultivated over a 2-year cycle, i.e., one year of vegetative growth followed by a fruit production year. After fruit harvesting, the stand is then mowed at the end of fall or early in the following spring.

### Experimental design

**Fig 1:**
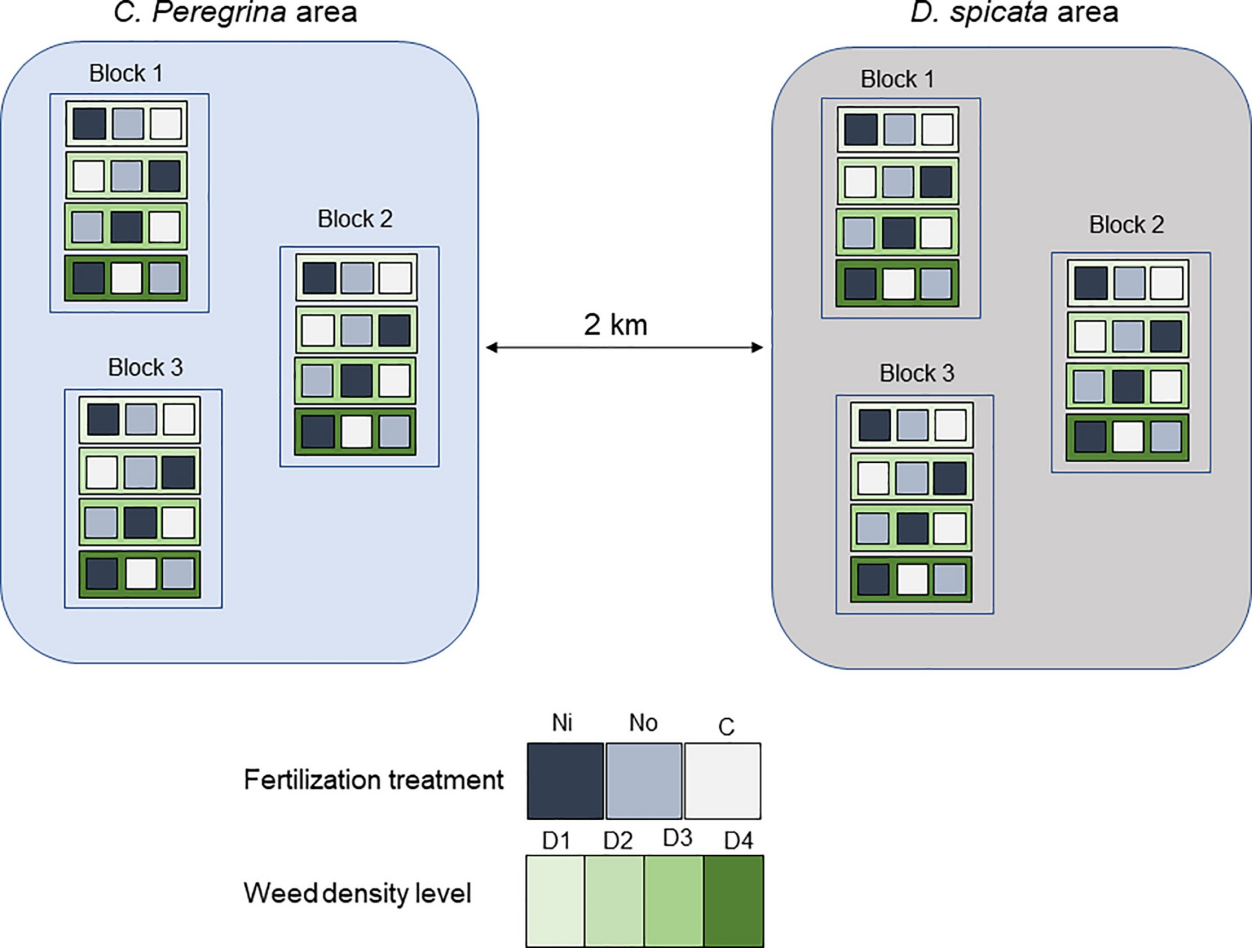
Schematic view of the experimental field. The studied lowbush blueberry commercial field contains two distinct areas (one colonized by *D*. *spicata* and the other one by *C*. *peregrina*) located ca. two kilometers away from each other. Each of these areas contain three blocks with four weed density levels and three N-fertilization treatment.

In autumn 2014, we surveyed the commercial field to find areas colonized either by *C*. *peregrina* or *D*. *spicata*. Two distinct areas separated by ca. 2 km (one colonized by *C*. *peregrina*, the other by *D*. *spicata*) were selected. In each area, we estimated initial weed density (number of plants·m^-2^) visually, and we established 1 × 1 m plots to have four levels of plant density for each weed (0–1, 2–3, 4–5, and 6+ plants m^-2^ for *C*. *peregrina*, and 0–1, 5–10, 15–20, and 25+ plants m^-2^ for *D*. *spicata*). Each of the four weed density plots was replicated three times—one density plot for each treatment—within three separate blocks (Fig 1). The blocks were located at least 100 m away from each other. We dug a narrow trench (~40 cm deep) around each experimental plot and filled it with two layers of polyethylene tarpaulin in order to isolate the root systems and retain the maximum amount of fertilizer within the plots. We then mowed the vegetation (lowbush blueberry and weeds) to a height of ~0.5 cm above the soil level in all 72 plots (2 weed species × 4 weed density levels × 3 fertilization treatments × 3 blocks).

We applied the fertilization treatment in early June 2015. For the inorganic N-fertilization treatment, we spread a 2 L solution of ammonium sulfate [(NH_4_)_2_SO_4_], which corresponded to an input of 45 kg N ha^-1^, i.e., the recommended N-fertilization rate for the region. As recommended in Quebec’s wild blueberry production guide, we also applied 20 kg ha^-1^ of P_2_O_5_ and 20 kg ha^-1^ K_2_O. For the organic N-fertilization, we applied composted CRW provided by the Ferland-Boileau Forestry Coopérative (Saguenay, Quebec) as a homogeneous layer. The CRW was composed exclusively of hardwoods, mainly fire cherry (*Prunus pennsylvanica*), green alder (*Alnus viridis*), and white birch (*Betula papyrifera*). Assuming a N-use efficacy of 12.5% for this type of organic material [24], equivalent to ca. 45 kg N ha^-1^, we required the application of 10 kg of CRW (wet basis) per plot, i.e., 100 t ha^-1^. This CRW input also provided the equivalent of ca. 22 kg P ha^-1^ and ca. 51 kg P ha^-1^. The chemical composition of the CRW is presented in the supplementary materials (S1 Table). After adding the CRW, we added 2 L of demineralized water. The control plots received no N-fertilization but received 20 kg ha^-1^ of P_2_O_5_ and 20 kg ha^-1^ K_2_O and 2 L of demineralized water. No pesticides or weed control (herbicides, mechanical control) were applied in any of the treatments.

### Blueberry foliar chemistry

In mid-July 2015, i.e., about two months after the application of the fertilizer, we harvested leaves from ten randomly selected stems from each plot. We dried the leaves at 60°C for 72 h and ground them using a cutting mill (Pulverisette 19, Fritsch, Idar-Oberstein, Germany). Chemical analyses were performed at Agriculture Canada’s experimental farm in Normandin after moist digestion with H_2_SO_4_ + H_2_O_2_ [25]. Colorimetry (Lachat Instruments, Quickchem Method 13-107-06-2-E; 15-501-03) was used to measure N and P concentrations, whereas K concentrations was determined by flame emission spectroscopy, and Ca and Mg concentrations by atomic absorption spectrometry (Perkin Elmer Analyst 300, Uberlingen, Germany).

### Blueberry fruit biomass

We hand-harvested the blueberry fruits from each plot during the week of August 15, 2016, and we weighed the fruits immediately after their collection. We removed a ~125 mL subsample of fruit for each plot and dried them at 65°C for five days; we used these samples to estimate the dry matter content. We estimated the mass of individual berries of this subsample by dividing the total mass by the number of berries contained in the subsample (78–538 berries).

### Total vegetative aboveground biomass

At the end of the production year (August 2016), we harvested the vegetative biomass of the weed species and lowbush blueberry from each plot—at ~0.5 cm above the soil level—and transported the harvested biomass to the lab. For each plot, we dried the aboveground biomass of each species at 60°C for 72 h and then measured the dry mass.

### Litter decomposition

We measured litter decomposition using the litterbag method. For this purpose, we manufactured 10 cm × 10 cm fiberglass net bags (1.5 mm mesh) that we filled with 2–3 g of fresh lowbush blueberry leaves. A fraction of the fresh litter was stored in the freezer (-14°C), another was dried at 55°C for 48 h to determine the water content of the fresh litter, and the other was buried within the litter bags in the plots on June 23, 2015, approximately one month after the application of the fertilizers. We buried three bags per plot, 30 cm apart. We placed the bags horizontally into the organic horizon of the soil, at 2–5 cm depth. We buried all three bags per plot along the same edge of the plots to minimize any disturbance to the existing biomass of the plots.

We placed fresh litter bags only in plots of the lowest and highest weed density, i.e., D1 and D4. During the experiment, we removed bags (one bag per plot) on three separate dates to assess the impact of incubation duration on litter decomposition. As such, we buried a total of 108 bags (3 fertilization treatments × 2 weed species × 2 weed density levels × 3 dates × 3 replicates = 108 bags). We collected one-third of the bags (36) in the fall of 2015 after 114 days of incubation. We removed another set of bags (36) from the soil in the spring of 2016 after 330 days of incubation, and the remaining bags were removed from the soil in August 2016 after 420 days of incubation.

Once the bags had been exhumed, we placed them immediately into plastic bags and stored the samples in a freezer (-14°C). To prepare the buried litter bags, we removed the litter from the bags, dried the litter at 55°C for 48 h, and then brushed away sand and root fragments from the litter samples using a binocular microscope. We then weighed and finely ground the samples (Mixer Mill MM 200, Retsch, Haan, Germany) before sending the material to Agriculture Canada's Normandin Experimental Farm for determining their respective C and N contents via dry combustion (TruMac CNS macro analyze, Leco, St. Joseph, USA).

We calculated the mass as well as N and C losses (%) during field incubation as:

$$\%\mathrm{loss}=\left[\left(X_{i}-X_{f}\right)/X_{i}\right]\times 100$$ Eq 1

where X_i_ and X_f_ are respectively the initial and final values for mass (g), C (g), or N (g) content of the samples.

### Statistical analyses

We performed a principal component analysis (PCA) on nine response variables and the 72 plots using the *ade4* package in R [26]. We then ran mixed-ANOVA on the number and mass of fruits produced by lowbush blueberry as well as the aboveground vegetative biomass production. The mixed models had fertilization treatment (three levels), weed species (two levels), and weed density (four levels) as fixed effects and blocks (three blocks per area) as the random effect. We also applied mixed-model analyses to litter decomposition with treatment (three levels), weed species (two levels), weed density (four levels), and incubation duration (three levels) as fixed effects and blocks (three blocks per site) as the random effect. Mixed-model analyses were performed running the *lmerTest* package in R, which uses the Satterthwaite's degrees of freedom method [27]. The package *emmeans*, which is an updated version of the *lsmeans* package [28], was used to estimate marginal means and conduct a post-hoc mean comparison between the factors’ levels.

## Results

### Blueberry fruit production

The analyses of variance revealed significant effects of the companion weed species, the N-fertilization treatments, and weed density on both the number and the mass of fruit produced per hectare (Table 1).

**Table 1:** Results of the mixed-model analyses for the tested variables of lowbush blueberry. Tested variables are fruit yield (t ha^-1^), fruit production (number of fruits ha^-1^), average individual fruit mass (mg), aboveground vegetative biomass (AGVBM, g m^-2^) and weed aboveground biomass (AGBM, g m^-2^). Fertilization treatment, weed species, and weed density were used as fixed effects, and “blocks” was used as a random effect. Significant effects are shown in bold (*P*<0.05).

		Fruit yield	Fruit number	Fruit mass	LB AGVBM	Weed AGBM
	Df	*P*	*P*	*P*	*P*	*P*
Fertilization	2	**1.5 10**^**−4**^	**6.3 10**^**−5**^	0.42	**1.8 10**^**−4**^	0.43
Weed species	1	**9.5 10**^**−8**^	**1.5 10**^**−5**^	**2.8 10**^**−5**^	**5.1 10**^**−11**^	**3.8 10**^**−16**^
Weed density	3	**1.3 10**^**−7**^	**1.5 10**^**−6**^	0.11	0.18	**6.3 10**^**−11**^
Fertilization × Weed	2	0.08	0.09	0.81	0.93	0.66
Fertilization × Weed density	6	0.25	0.41	0.40	0.78	0.64
Weed species × Weed density	3	0.08	0.28	0.96	**3.4 10**^**−6**^	**5.7 10**^**−4**^
Fertilization × Weed species × Weed density	6	0.76	0.82	0.51	0.89	0.78

**Fig 2:**
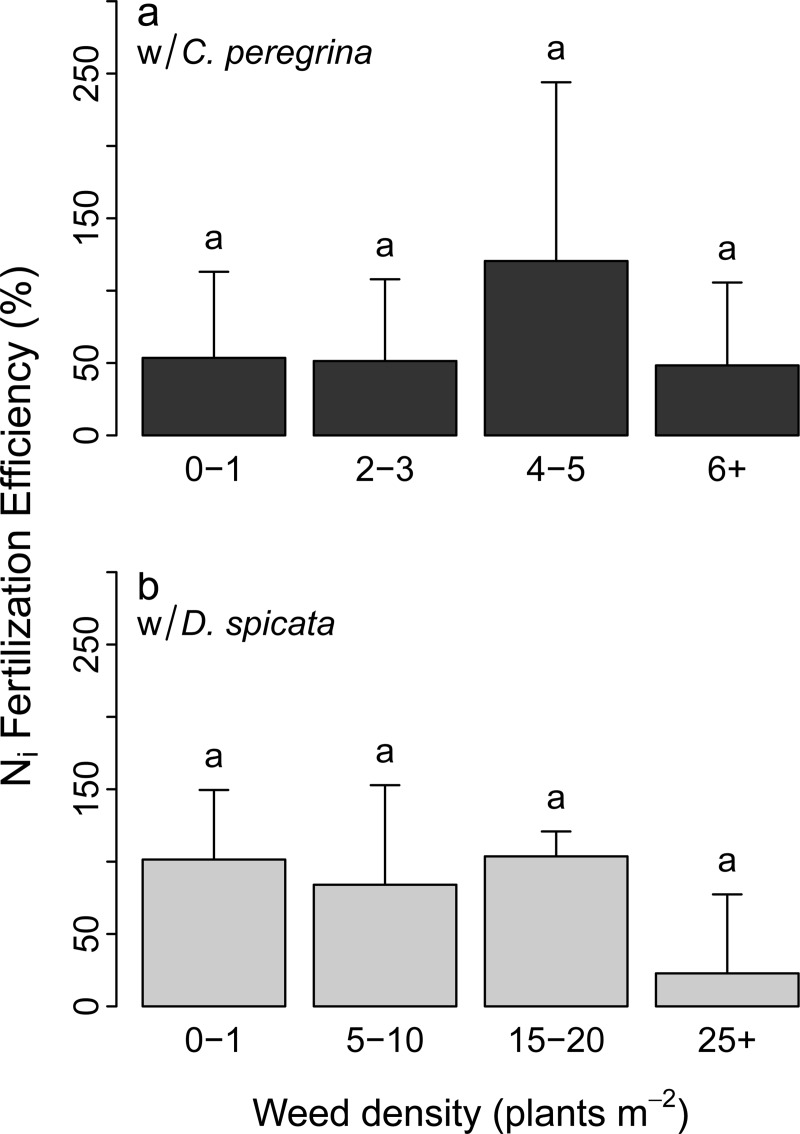
Increased fruit yield (%) due to the inorganic N treatment (inorganic N-fertilization efficiency) as a function of weed density. The increase in fruit yield was determined relative to fruit yield in control plots. (a) Inorganic N-fertilization efficiency in plots of *C*. *peregrina*; (b) Inorganic N-fertilization efficiency in plots of *D*. *spicata*. Values are mean ± SD (*n* = 3). Values that share the same letters above the bar plots are not significantly different (*P* > 0.05).

Plots having *D*. *spicata* produced fruit yields that were almost twice that of those having *C*. *peregrina* (6.3 vs. 3.2 t ha^-1^; Table 2). On average, fruit yield was 48% higher in the inorganic N-fertilization plots (6.3 ± 4.6 t ha^-1^) than in the organic N-fertilization (4.3 ± 2.8 t ha^-1^) and 71% higher than in the control plots (3.7 ± 2.3 t ha^-1^). Although not statistically significant, the application of CRW tended to increase fruit yield by 300 and 800 kg ha^-1^ for *C*. *peregrina* and *D*. *spicata*, respectively, compared to the control plots (Table 2). The efficiency of inorganic N-fertilization (%), i.e. the factor by which N-fertilization increased fruit yield, did not differ significantly between *C*. *peregrina* and *D*. *spicata* plots or between density levels (Fig 2). Nonetheless, the efficiency of inorganic N-fertilization was: i) 2× higher in *D*. *spicata* plots (100%) than in *C*. *peregrina* plots (50%) at low weed density levels (D1 and D2); ii) 2× lower in high-density plots (D4) with *D*. *spicata* than with *C*. *peregrina*; and iii) 4× times lower (25%) in *D*. *spicata* plots at high density (>25 plants m^-2^) than at lower density levels (0–1, 5–10, and 15–20 plants m^-2^).

**Table 2:** Average values (± SD) of the measured variables for blueberry and weeds in plots colonized by *C*. *peregrina* and *D*. *spicata* subjected to the three fertilization treatments. Measured variables included lowbush blueberry fruit yield (t ha^-1^), number of fruits produced (10^6^ ha^-1^), individual fruit mass (mg), aboveground vegetative biomass (AGVBM; g m^-2^), fruit yield: aboveground vegetative biomass ratio, and weed aboveground biomass (AGBM; g m^-2^) under the three N-fertilization treatments in plots having *C*. *peregrina* or *D*. *spicata* as companion weed species. Values not sharing the same letters are significantly different (*P* <0.05). Roman letters are used to compare N-fertilization treatments, whereas Greek letters are used to compare the two areas of the field (i.e., *C*. *peregrina* area vs. *D*. *spicata* area).

	*C*. *peregrina* area				*D*. *spicata* area			
	Inorganic N	Organic N	Control	Mean	Inorganic N	Organic N	Control	Mean
Fruit yield (t ha^-1^)	4.0 ± 1.8 (a)	3.0 ± 1.4 (b)	2.7 ± 1.5 (b)	3.2 ± 1.6 (α)	8.6 ± 5.4 (a)	5.5 ± 3.3 (b)	4.7 ± 2.7 (b)	6.3 ± 4.2 (β)
No. of fruits (10^6^ ha^-1^)	18.6 ± 8.9 (a)	12.6 ± 4.9 (b)	10.9 ± 4.7 (b)	14.0 ± 7.1 (α)	32.4 ± 21.2 (a)	19.0 ± 10.7 (b)	15.2 ± 8.7 (b)	22.2 ± 16.0 (β)
Individual fruit mass (mg)	228 ± 62 (a)	233 ± 50 (a)	238 ± 49 (a)	233 ± 53 (α)	275 ± 46 (a)	297 ± 71 (a)	305 ± 48 (a)	292 ± 56 (β)
AGVBM (g m^-2^)	392 ± 102 (a)	331 ± 64 (ab)	301 ± 67 (b)	341 ± 86 (α)	254 ± 92 (a)	203 ± 106 (a)	176 ± 96 (a)	211 ± 101 (β)
Yield: AGVBM ratio	1.1 ± 0.7 (a)	0.9 ± 0.4 (a)	0.9 ± 0.5 (a)	1.0 ± 0.2 (α)	3.5 ± 2.1 (a)	3.1 ± 2.1 (a)	3.0 ± 1.9 (a)	3.2 ± 1.0 (β)
Weed AGBM (g m^-2^)	268 ± 171 (a)	225 ± 101 (a)	241 ± 143 (a)	245 ± 138 (α)	54 ± 60 (a)	46 ± 62 (a)	48 ± 57 (a)	49 ± 58 (β)

**Fig 3:**
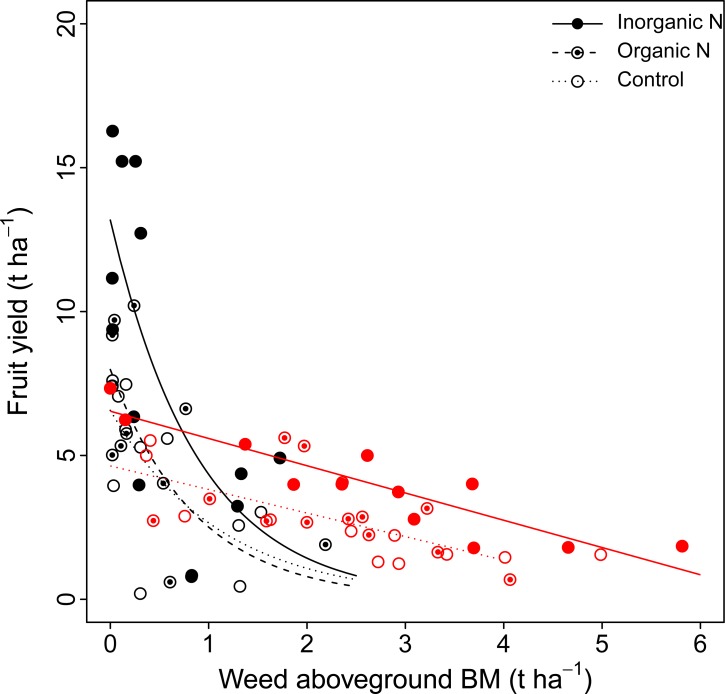
Relationship between lowbush blueberry fruit yield (t ha^-1^) and the aboveground biomass of weeds (t ha^-1^). Values are shown for *C*. *peregrina* (red symbols) and *D*. *spicata* (black symbols) under the three fertilization treatments (inorganic N, organic N, and control). Curves show significant relationships (*P* < 0.05). *C*. *peregrina* plots–Inorganic N: y = 6.53–0.94x (*R*^2^ = 0.82); Control: y = 4.63–0.82x (*R*^2^ = 0.63). *D*. *spicata* plots–Inorganic N: y = 13.18e^1.11x^ (*R*^2^ = 0.51); Organic N: y = 7.99e^1.16x^ (*R*^2^ = 0.51); Control: y = 6.57e^0.90x^ (*R*^2^ = 0.45).

Fruit yield decreased with increasing weed aboveground biomass under all fertilization treatments (Fig 3), illustrating the detrimental effect of weed density on fruit yield (Table 1; S1 Fig). This fruit yield–weed aboveground biomass decrease was linear for *C*. *peregrina* plots, whereas it was best described by a decreasing exponential function (y = ae^-bx^) for the *D*. *spicata* plots. The number of fruits produced per hectare was highest in plots having *D*. *spicata* fertilized with inorganic N (Table 2). The number of fruits produced per hectare also decreased with increasing weed density (S2 Fig). In contrast, N-fertilization did not affect the average mass of individual berries (Table 1); however, the average mass of individual berries was significantly higher in plots having *D*. *spicata* than in those having *C*. *peregrina* (Table 2).

### Blueberry and weed vegetative biomass production

**Fig 4:**
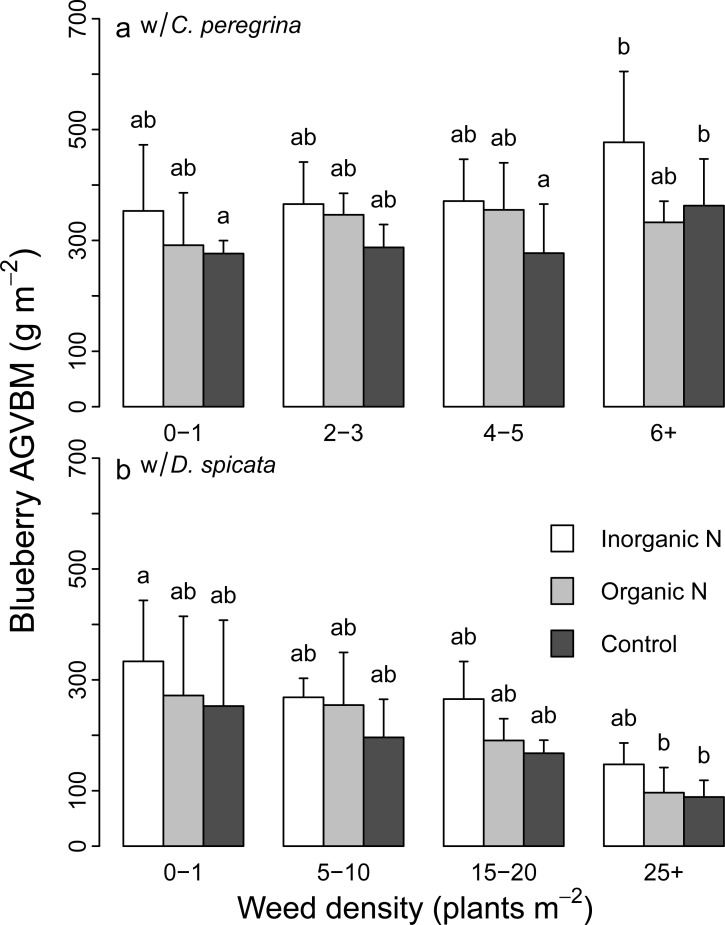
Lowbush blueberry aboveground vegetative biomass (AGVBM) at the end of the production cycle (g m^-2^). AGVBM values (mean ± SD) are shown for the three fertilization treatments (inorganic N-fertilization, organic N-fertilization, and control plots) and the four levels of weed density. (a) Lowbush blueberry AGVBM in plots of *C*. *peregrina*; (b) lowbush blueberry AGVBM in plots of *D*. *spicata*; bars of AGVBM values not sharing the same letters are significantly different (*P* < 0.05).

The N-fertilization treatments and the companion weed species had a significant effect on the aboveground vegetative biomass of blueberry over the two years of the experiment (Table 1). Blueberry aboveground vegetative biomass was 30–40% higher in the inorganic N-fertilization plots relative to the control plots, regardless of the competing weed species (Table 2). In contrast, N-fertilization did not affect weed biomass (Table 1). Weed biomass was much higher in *C*. *peregrina* plots than in *D*. *spicata* plots under all N-fertilization treatments (Table 2). The significant interaction between weed species and weed density (Table 1) reflects the decrease in blueberry aboveground vegetative biomass with increased *D*. *spicata* density (Fig 4B), whereas no such trend was observed in plots having *C*. *peregrina* (Fig 4A). The fertilization treatments did not significantly affect the blueberry fruit yield: aboveground vegetative biomass ratio. In contrast, this ratio was significantly higher in plots having *D*. *spicata* than those with *C*. *peregrina* for all fertilization treatments (Table 2).

### Blueberry foliar chemistry

The N-fertilization treatments did not significantly affect blueberry foliar N, P, K, Mg, and Ca concentrations. In contrast, there was a significant difference in N, Mg, and Ca concentrations between the area colonized by *C*. *peregrina* and the one colonized by *D*. *spicata* (Table 3). On average, blueberry foliar N and Mg concentrations were higher in plots having *C*. *peregrina* than in those having *D*. *spicata* (16.5 and 15.3 mg g^-1^, respectively, for N concentrations, and 1.8 and 1.7 mg g^-1^, respectively, for Mg concentrations). On the contrary, the foliar Ca concentration of blueberry was higher in plots having *D*. *spicata* than in those having *C*. *peregrina* (5.1 and 4.5 mg g^-1^, respectively).

**Table 3:** Foliar chemistry of lowbush blueberry. Concentrations of N, P, K, Ca, and Mg (mg g^-1^) in plots having *C*. *peregrina* or *D*. *spicata* as companion species of lowbush blueberry. Values are mean ± SD. Values not sharing the same letters are significantly different (*P* < 0.05). Roman letters are used to compare N-fertilization treatments, whereas Greek letters are used to compare the two areas of the field (i.e., *C*. *peregrina* area vs. *D*. *spicata* area).

	*C*. *peregrina* area				*D*. *spicata* area			
	Inorganic N	Organic N	Control	Mean	Inorganic N	Organic N	Control	Mean
N	16.6 ± 1.0 (a)	16.6 ± 1.0 (a)	16.4 ± 0.7 (a)	16.5 ± 0.9 (α)	15.1 ± 0.9 (a)	15.7 ± 1.4 (a)	15.1 ± 0.9 (a)	15.3± 1.1 (β)
P	1.2 ± 0.1 (a)	1.3 ± 0.1 (a)	1.2 ± 0.1 (a)	1.2 ± 0.1 (α)	1.3 ± 0.1 (a)	1.2 ± 0.1 (a)	1.3 ± 0.1 (a)	1.3 ± 0.1 (α)
K	5.8 ± 0.5 (a)	5.6 ± 0.4 (a)	5.6 ± 0.5 (a)	5.6 ± 0.5 (α)	5.9 ± 0.9 (a)	5.6 ± 0.7 (a)	6.0 ± 0.7 (a)	5.8 ± 0.8 (α)
Ca	4.3 ± 0.8 (a)	4.6 ± 0.7 (a)	4.7 ± 0.5 (a)	4.5 ± 0.7 (α)	5.1 ± 0.5 (a)	4.9 ± 0.5 (a)	5.4 ± 1.0 (a)	5.1 ± 0.7 (β)
Mg	1.8 ± 0.2 (a)	1.8 ± 0.2 (a)	2.0 ± 0.2 (a)	1.8 ± 0.2 (α)	1.6 ± 0.2 (a)	1.7 ± 0.3 (a)	1.8 ± 0.4 (a)	1.7 ± 0.3 (β)

**Fig 5:**
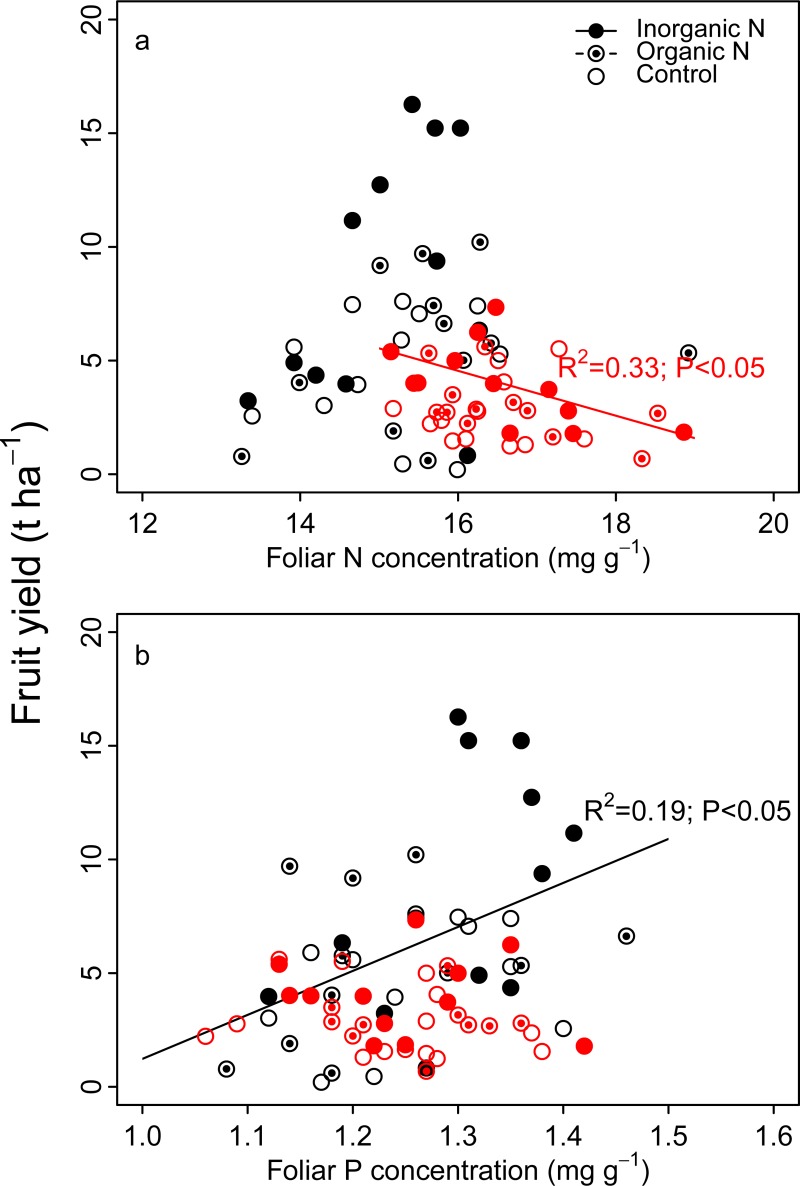
Relationships between lowbush blueberry foliar chemistry and fruit yield (t ha^-1^). (a) Relationship between N concentrations (mg g^-1^) and fruit yield; (b) Relationship between P concentrations (mg g^-1^) and fruit yield. Results are shown for plots of *C*. *peregrina* (red symbols) and *D*. *spicata* (black symbols) subjected to the three fertilization treatments (inorganic N, organic N, and control).

There was a significant negative relationship between blueberry foliar N concentration and fruit yield in plots having *C*. *peregrina* as a companion weed species and an inorganic N-fertilization treatment (Fig 5A). No significant relationship was found between these two variables in plots having *D*. *spicata*. There was a positive relationship between fruit yield and foliar P concentration in association with *D*. *spicata* for N-fertilization treatments (Fig 5B). We found no such relationship in plots having *C*. *peregrina*.

**Fig 6:**
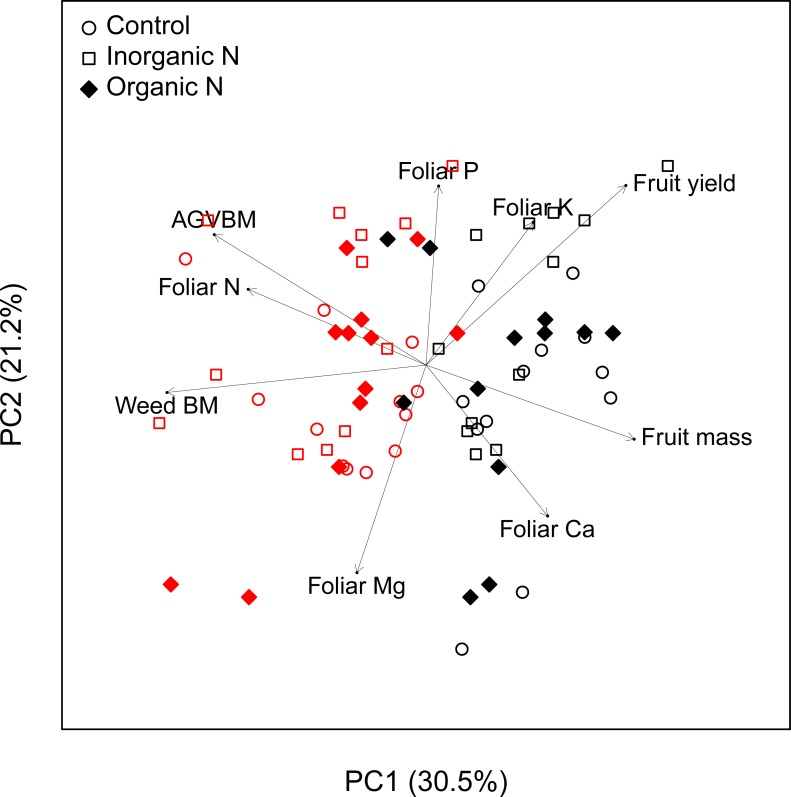
Principal component analysis of *C*. *peregrina* and *D*. *spicata* plots. *C*. *peregrina* (red symbols) and *D*. *spicata* (black symbols) are presented with the variables of blueberry aboveground vegetative biomass (AGVBM; t ha^-1^), fruit yield (t ha^-1^), individual fruit mass (mg), weed aboveground biomass (Weed BM; t ha^-1^), and foliar concentrations of N, P, K, Ca, and Mg (mg g^-1^).

A principal component analysis (PCA) summarizes the influence of fertilization and the presence of weeds on lowbush blueberry (Fig 6). Plots having *C*. *peregrina* (red symbols) or *D*. *spicata* (black symbols) were well separated on the first axis (PC1), whereas plots were not separated as a function of the N-fertilization treatment they received. Plots having *C*. *peregrina* (red symbols) were associated with a higher weed aboveground biomass and a higher blueberry aboveground vegetative biomass (AGVBM) as well as higher foliar N concentrations. By contrast, plots with *D*. *spicata* (black symbols) were associated with higher blueberry fruit yields, foliar Ca concentration, and individual fruit mass.

### Litter decomposition

**Fig 7:**
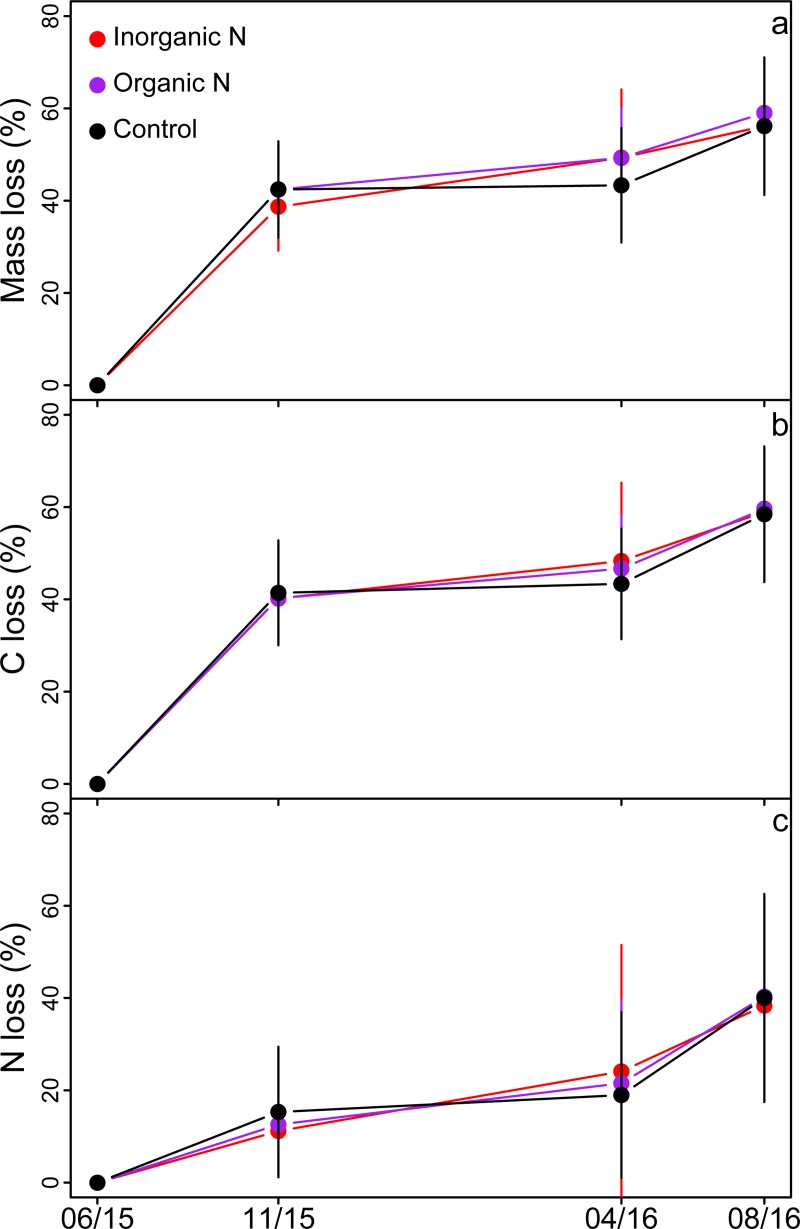
Impact of the fertilization treatments on litter decomposition. (a) Mass loss (%), (b) C loss (%), and (c) N loss (%) from the litter 114, 330, and 420 days after the litter bags were buried in the three experimental plots (inorganic N, organic N, and control plots).

None of the studied variables (N-fertilization treatments, weed species, and weed density) affected the measured litter decomposition variables, namely total mass, C and N losses (ANOVA; data not shown). Litter mass and C content decreased by 40%, and N loss by 15% during the first 114 days of incubation, between June and November 2015 (Fig 7). Mass, C, and N losses were not significant during the following winter (from December to April). Litter mass, C, and N content decreased by an additional 10–15% from May to August 2016 (Fig 7).

## Discussion

### Effect of N-fertilization on plant biomass production and foliar chemistry

Most plants species, especially in cold ecosystems, can fulfill a portion of their N demand by taking up small organic molecules, e.g., amino acids and proteins, from the soil [8,29–33]. Ericaceous species are particularly efficient in taking up N from recalcitrant soil organic matter [10,34,35] due to their ericoid mycorrhizal associations [36–38]. We therefore expected the organic N treatment of CRW to have a positive effect on lowbush blueberry biomass production as reported in a recent review [12]. Although slight increases were observed, the organic N-fertilization treatment did not significantly increase blueberry aboveground vegetative biomass production or fruit yield relative to the unfertilized controls. This lack of effect did not result from competition for N with weed species as the weeds’ biomass did not increase with the organic N-fertilization treatment either. As CRW not only provides nutrients but also improves soil conditions in the long term—moisture, structure, biological activity, etc.—the absence of an effect may be due to the short duration of the experiment. A beneficial effect may require a number of years of application before appearing. That organic N-fertilization did not stimulate *D*. *spicata* biomass agreed with our expectations as Poaceae species rely mainly upon inorganic N [39]. A previous ^15^N-labelling study has shown that *D*. *spicata* specifically relies heavily on inorganic N fertilizer for producing its aboveground biomass [7]. The absence of an effect for the organic N fertilizer on the three plant species may have resulted from the specific organic fertilizer used. First, CRW has a high C:N ratio (61; S1 Table), which can decrease inorganic N availability by stimulating N immobilization the year following CRW application [12,40,41]. Second, the ~2 cm thick layer of CRW (10 kg of RCW per m^2^) may have impeded vegetative growth by changing soil physicochemical conditions, e.g., moisture and temperature, through its mulching effect. The latter explanation seems unlikely, however, as adding CRW compost did not modify soil litter decomposition in our study.

The inorganic N-fertilization did not enhance weed biomass production. Although it was not entirely surprising for *C*. *peregrina*, which can use atmospheric N through an actinorhizal association, the lack of enhanced biomass production was more surprising for *D*. *spicata*, which is very effective in taking up inorganic N derived from fertilizer due to its dense and superficial root system [7]. By contrast, the inorganic N-fertilization treatment significantly enhanced blueberry fruit yield and aboveground vegetative biomass, regardless of the associated weed species. Estimations of aboveground vegetative biomass using the «point intercept method» [42] showed that the vegetative growth was particularly stimulated the year following fertilizer application (S3 Fig). This result agrees with several studies that report increased blueberry nutritional status, growth [6,43–46], and fruit production after repeated additions of inorganic N [6,46–48]. As well, the inorganic N-fertilization treatment enhanced fruit yield more than aboveground vegetative biomass production, resulting in a 15–20% higher fruit yield to aboveground vegetative biomass ratio in the inorganic N plots relative to the organic N and control plots (Table 2). The positive effect of the inorganic N treatment on fruit yield resulted mainly from an increase in the number of berries produced, supporting results from a previous study [49]. The fertilization treatment had no significant effect on the mass of individual berries, showing that berry mass was not limited by N availability. Much of the observed increase in fruit yield likely resulted from an increase in the number of flowers per surface area as reported in another study [46]. Our observations may be, however, also related to decreased fruit abortion. Penney et al. [49] observed that the number of living flower buds did not differ between the fertilized and unfertilized plots. They speculated that the increased number of berries following N-fertilization stemmed from lower fruit abortion. More research is required to clarify this point.

As *D*. *spicata* relies heavily on inorganic N and the presence of this species can be detrimental for blueberry vegetative biomass production, especially at high-density levels [7], we expected a lower inorganic-N fertilizer efficiency in *D*. *spicata* than in *C*. *peregrina* plots. This hypothesis was not supported by our data, although this efficiency tended to be lower at high *D*. *spicata* density. Whereas the inorganic N-fertilization treatment increased fruit yield by ca. 100% in plots having density levels lower than 15–20 plants m^-2^, the increase was only 20% in plots with >25 plants m^-2^ (Fig 2B). Therefore, inorganic N-fertilization may not be a good strategy for farmers when *D*. *spicata* density exceeds 25 plants m^-2^.

Contrary to other studies, N-fertilization had no significant effect on foliar N concentrations of blueberry. This absence of increased N foliar concentration may have resulted from a dilution effect because blueberry aboveground biomass production increased following inorganic-N-fertilization. A large fraction of N derived from the fertilizer may also have been allocated to fruit production rather than to the vegetative parts. Overall, the range of foliar N concentration reported here (13.3–18.9 mg g^-1^) is slightly lower than the ones reported for other fertilized commercial fields [50–53]. This likely results from the comparatively young age of our study field (cultivated since 2005) and the moderate use of fertilizers.

### Impact of weeds on blueberry biomass production and foliar N concentration

The PCA (Fig 6) highlighted the stronger influence of the companion weed species than the type of fertilization treatment on blueberry variables. The strong separation of plots colonized by *C*. *peregrina* and *D*. *spicata* reflected their respective and distinct impacts on blueberry crops. Although both weed species affected blueberry aboveground vegetative biomass and fruit yield, the weed species differed in their impact on blueberry production. *D*. *spicata* had a greater negative impact on vegetative biomass than on fruit yield. Conversely, *C*. *peregrina* negatively impacted fruit yield more than vegetative biomass. The aboveground vegetative biomass production of blueberry was 40% lower in plots colonized by *D*. *spicata* than in those colonized by *C*. *peregrina*, whereas blueberry fruit production in plots having *C*. *peregrina* was half that of plots having *D*. *spicata*, regardless of the N-fertilization treatment (Table 2). This pattern indicates a decoupling between vegetative and fruit biomass as shown by the lack of relationship between these two variables regardless of the fertilization treatment or weed species (*r* = -0.10; *P* = 0.38). Higher vegetative growth in plots having *C*. *peregrina* has been shown to result from the ability of *C*. *peregrina* to derive N from the atmosphere, especially when present at high density, thus alleviating the competition for soil N acquisition [7]. Differences in fruit yield between plots colonized by *C*. *peregrina* and *D*. *spicata* was more driven by the number of fruits per surface area than by the mass of individual berries. The number of fruits per hectare in plots having *D*. *spicata* was ca. 2× that of *C*. *peregrina* plots (22.2 10^6^ vs. 14.0 10^6^ ha^-1^, respectively), whereas the mass of individual berries was only 25% higher in *D*. *spicata* plots (292 ± 56 mg vs. 233 ± 53 mg, respectively). The reasons for such a difference are unclear. The predominance of one of the two weed species may have impacted blueberry flower production, pollination efficiency, fruit abortion or any other mechanisms involved in fruit production. For instance, *C*. *peregrina*, as other species in the Myricaceae family, produces strong odors, insect-repellant secondary substances [54,55], and cytotoxic molecules [56] that can repulse insect pollinators, and could thus decrease flower visitation rates by pollinators and pollination success in nearby blueberry patches. Previous studies and personal observations also suggest that *C*. *peregrina* may reduce light availability in blueberry patches, as *C*. *peregrina* grows taller than both blueberry and *D*. *spicata*. Although lowbush blueberry plants can persist under a canopy, at least 50% full sunlight is required for flower bud formation and fruiting [57,58]. Shade from taller weeds can reduce fruit yields when sunlight is reduced below 80% [5,59]. Further studies are necessary to address these possibilities. However, our data revealed an “area effect” on fruit yield. Indeed, fruit yields were higher in *D*. *spicata* plots even in low weed density plots (D1 level), in which weed species were almost absent (weed aboveground biomass null or close to zero; Fig 3). This occurred although the commercial field is very homogeneous and the two selected areas (the one colonized by *C*. *peregrina* and the other by *D*. *spicata*) are situated only ca. 2 km away from each other. In addition, available soil analyses for the site show minimal differences between the two areas. A “population effect” may be responsible for the difference between the two areas as several studies report a large variability in fruit yields among clones and species as well as a large influence of the degree of cross-pollination and self-fertility in blueberry species [60–62]. Another hypothesis is an excessive amount of foliar N in plots colonized by *C*. *peregrina* (Table 3), which can be detrimental to fruit production in blueberry [50]. This hypothesis is supported by the negative relationship between blueberry foliar N concentrations and fruit production in the plots having *C*. *peregrina* (Fig 5). This mechanism does not contradict the positive effect of inorganic N on fruit production in *C*. *peregrina* plots because inorganic N-fertilization did not significantly increase foliar N concentration (Table 3).

### Litter decomposition rate

The direction and magnitude of the effect of N-fertilization on litter decomposition vary between studies as this effect depends on several interacting factors, such as the rate and type of fertilizer applied, litter quality, moisture, and the rate of N deposition. A recent meta-analysis showed that litter decay response to N addition ranged from 38% inhibition to 64% stimulation depending on experimental conditions [63]. In studies where N inputs were < 75 kg N ha^-1^, there was a slight but significant decrease in litter decomposition rates (-5%). When N inputs were 75–125 kg N ha^-1^, there was a significant stimulation of litter decomposition (+17%). In our experiment, N addition (45 kg N ha^-1^)—either in an inorganic or organic form—did not significantly alter litter decomposition variables, i.e., loss of mass, C, and N. At the end of the 420-day incubation period, the litter had lost ~55% of its initial mass and C content and ~40% of its initial N content. Most litter mass and C loss (~40%) occurred during the first growing season, and the litter did not lose a significant fraction of mass, C, or N during the following winter (Fig 7), most likely because of the low winter temperatures that impeded soil microbial activity. Losses of litter mass and C in the second season were less than the first growing season as most of the labile fraction of the litter decomposed in the first growing season, making the litter more recalcitrant the second year [64].

## Conclusion

In contrast with our initial hypothesis, composted CRW did not significantly improve lowbush blueberry fruit yield nor vegetative biomass and foliar N concentration, suggesting that this type of organic fertilizer did not release a significant amount of readily available N in the time frame of the study. In contrast, the addition of inorganic N enhanced fruit yield by ~70% relative to the control plots. The beneficial effect of this fertilization treatment depended on the companion weed species and its density, revealing complex competition mechanisms likely driven by weed functional traits and local soil conditions. Although blueberry fruit yield was on average twice higher in plots having *D*. *spicata* than in plots having *C*. *peregrina*, blueberry growth and fruit yield as well as fertilization efficiency were more strongly impacted by increasing *D*. *spicata* than *C*. *peregrina* density. As shown in a previous study, this likely resulted from the ability of *C*. *peregrina* to derive some of its N from the atmosphere as its density increased [7]. A close analysis of the data also revealed that, in addition to weed competition, unknown local site conditions contributed to the variability in fruit yield at the field scale. Overall, the present study reveals the distinct interactions between lowbush blueberry and weed species and confirms the necessity of weed control to improve fruit yields and the efficiency of inorganic N-fertilization. We recommend keeping *D*. *spicata* density as low as possible to maximize fruit yields and under 25 plants m^-2^ to optimize inorganic N-fertilization efficiency.

## Supporting information

S1 Table Results of chemical analyses performed on the chipped ramial wood (CRW) used as organic fertilizer. (DOCX)

S1 Fig Lowbush blueberry fruit yield (t ha^-1^) as a function of the N-fertilization treatment and weed density. (a) Values in sweet fern plots; (b) Values in poverty oat grass plots. Values (mean ± SD) not sharing the same letter are significantly different (*P* < 0.05). See material and methods for details of the statistical analyses.
(DOCX)

S2 Fig Lowbush blueberry fruit yield (t ha^-1^) and number of fruits produced (10^6^ ha^-1^). (a–c) Fruit yield as a function of companion weed species, N-fertilization treatment, and weed density, respectively. (d–f) Number of fruits produced as a function of companion weed species, N-fertilization treatment, and weed density, respectively. Values (mean ± SD) not sharing the same letter are significantly different (*P* < 0.05). See material and methods for details of the statistical analyses.
(DOCX)

S3 Fig Aboveground vegetative biomass (AGVBM) (g m^-2^) estimated using the point intercept method. AGVBM values (mean ± SD) are shown for the three N-fertilization treatments over the two years of the production cycle. Asterisks indicate significant fertilization effects. (a) Lowbush blueberry; (b) sweet fern; (c) poverty oat grass (c).
(DOCX)

S4 Fig (JPG)

S1 Data (CSV)

S2 Data (CSV)
