# Supplementary material for: Lowbush blueberry fruit yield and growth response to inorganic and organic N-fertilization when competing with two common weed species
Source: PLoS One. 2019 Dec 26;14(12):e0226619. doi: 10.1371/journal.pone.0226619 (PMC6932764; doi:10.1371/journal.pone.0226619)
Supplement: S1 Table — (DOCX) [file pone.0226619.s001.docx]

**S1 Table. Results of chemical analyses performed on the chipped ramial wood (CRW) used as organic fertilizer.**

|  |  | Value |
| --- | --- | --- |
| N (mg g^-1^) |  | 1.77 |
| N-NH_4_ (mg g^-1^) |  | 0.00 |
| P (mg g^-1^) |  | 0.22 |
| P_2_O_5_ (mg g^-1^) |  | 0.51 |
| K (mg g^-1^) |  | 0.52 |
| Ca (mg g^-1^) |  | 2.62 |
| Mg (mg g^-^1) |  | 0.16 |
| Dry matter (%) |  | 22.39 |
| Organic matter (%) |  | 96.52 |
| pH |  | 6.39 |
| C:N ratio |  | 61.11 |
