# Supplementary material for: Lowbush blueberry fruit yield and growth response to inorganic and organic N-fertilization when competing with two common weed species
Source: PLoS One. 2019 Dec 26;14(12):e0226619. doi: 10.1371/journal.pone.0226619 (PMC6932764; doi:10.1371/journal.pone.0226619)
Supplement: S1 Fig — (a) Values in sweet fern plots; (b) Values in poverty oat grass plots. Values (mean ± SD) not sharing the same letter are significantly different (P < 0.05). See material and methods for details of the statistical analyses. (DOCX) [file pone.0226619.s002.docx]

**S1 Fig. Lowbush blueberry fruit yield (t ha^-1^) as a function of the N-fertilization treatment and weed density.**

(a) Values in sweet fern plots; (b) Values in poverty oat grass plots. Values (mean ± SD) not sharing the same letter are significantly different (*P* < 0.05). See material and methods for details of the statistical analyses.
