# Supplementary material for: Lowbush blueberry fruit yield and growth response to inorganic and organic N-fertilization when competing with two common weed species
Source: PLoS One. 2019 Dec 26;14(12):e0226619. doi: 10.1371/journal.pone.0226619 (PMC6932764; doi:10.1371/journal.pone.0226619)
Supplement: S2 Fig — (a–c) Fruit yield as a function of companion weed species, N-fertilization treatment, and weed density, respectively. (d–f) Number of fruits produced as a function of companion weed species, N-fertilization treatment, and weed density, respectively. Values (mean ± SD) not sharing the same letter are significantly different (P < 0.05). See material and methods for details of the statistical analyses. (DOCX) [file pone.0226619.s003.docx]

**S2 Fig. Lowbush blueberry fruit yield (t ha^-1^) and number of fruits produced (10^6^ ha^-1^).**

(a–c) Fruit yield as a function of companion weed species, N-fertilization treatment, and weed density, respectively. (d–f) Number of fruits produced as a function of companion weed species, N-fertilization treatment, and weed density, respectively. Values (mean ± SD) not sharing the same letter are significantly different (*P* < 0.05). See material and methods for details of the statistical analyses.
