# Supplementary material for: Lowbush blueberry fruit yield and growth response to inorganic and organic N-fertilization when competing with two common weed species
Source: PLoS One. 2019 Dec 26;14(12):e0226619. doi: 10.1371/journal.pone.0226619 (PMC6932764; doi:10.1371/journal.pone.0226619)
Supplement: S3 Fig — AGVBM values (mean ± SD) are shown for the three N-fertilization treatments over the two years of the production cycle. Asterisks indicate significant fertilization effects. (a) Lowbush blueberry; (b) sweet fern; (c) poverty oat grass (c). (DOCX) [file pone.0226619.s004.docx]

**S3 Fig. Aboveground vegetative biomass (AGVBM) (g m^-2^) estimated using the point** **intercept method.**

AGVBM values (mean ± SD) are shown for the three N-fertilization treatments over the two years of the production cycle. Asterisks indicate significant fertilization effects. (a) Lowbush blueberry; (b) sweet fern (*C. peregrina*); (c) poverty oat grass (*D. spicata*) (c).


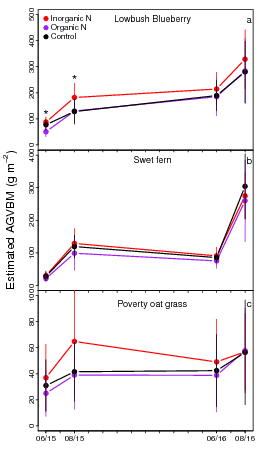


We used the “point intercept method” to estimate the vegetative aboveground biomass of each plant species. We estimated this aboveground biomass for plant cover twice a year (June and August) during the first and second years of the experiment (2015 and 2016). The point intercept method is a reliable, efficient, inexpensive, and fast method for estimating the vegetative aboveground biomass of poverty oat grass, sweet fern, and lowbush blueberry in commercial fields. This method consists of placing a grid pattern over a sampling quadrat and determining the plant species found at the interception of each grid point. Lévesque et al..(2018) showed a very strong correlation between the number of point intercepts and vegetative aboveground biomass for the three study species (0.76 < *R*^2^ <0.88). In our study, we used the calibrated regression parameters from Lévesque et al. (2018) to estimate vegetative aboveground biomass for each species through the first and second years.

Lévesque J, Bradley RL, Bellemare M, Lafond J, Paré MC. Predicting weed and lowbush blueberry biomass using the point intercept method. Can J Plant Sci. 2018;4:1–4.
