# Supplementary figures and images for: Lowbush blueberry fruit yield and growth response to inorganic and organic N-fertilization when competing with two common weed species
Source: PLoS One. 2019 Dec 26;14(12):e0226619. doi: 10.1371/journal.pone.0226619 (PMC6932764; doi:10.1371/journal.pone.0226619)

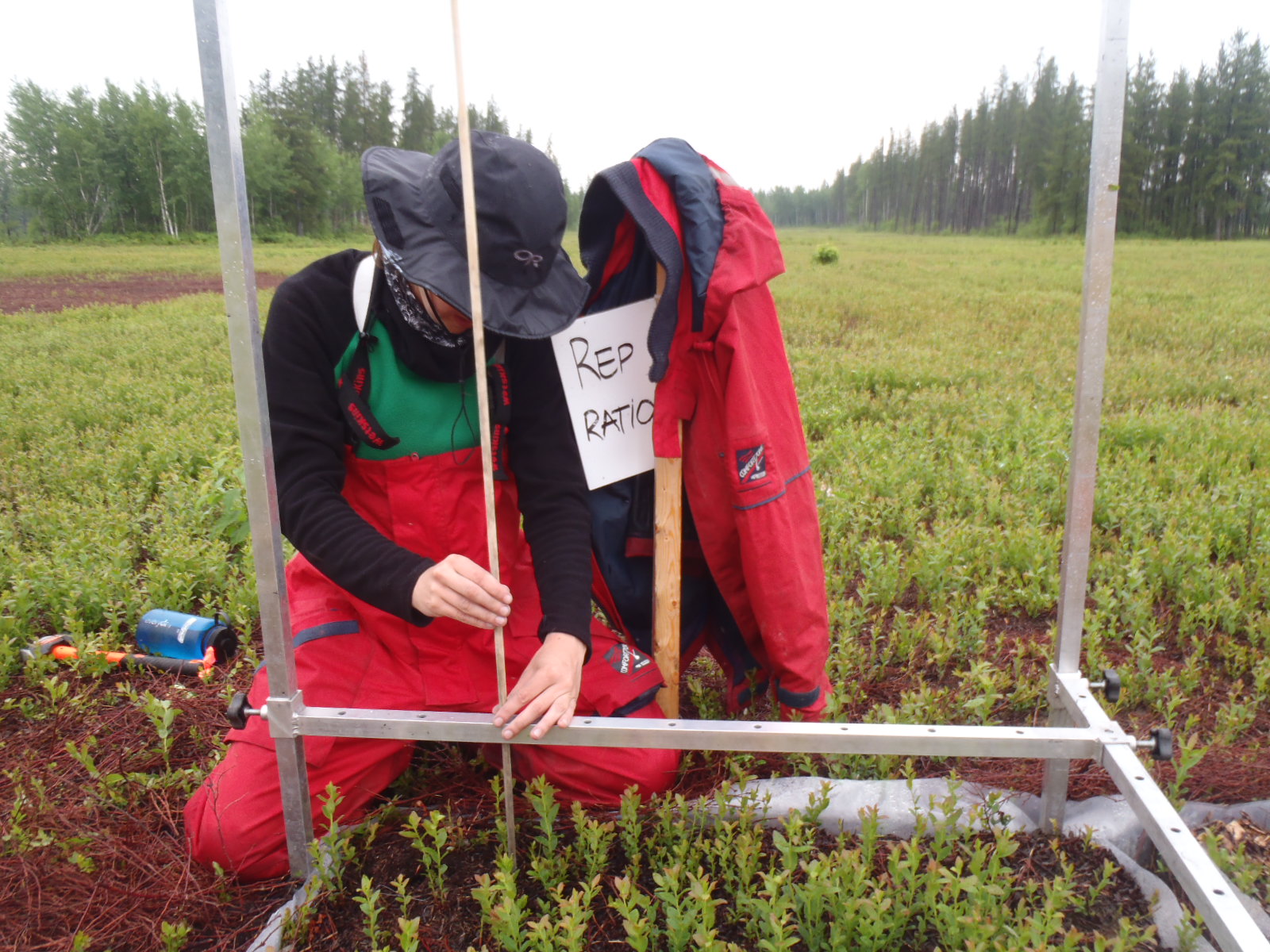

Supplement: S4 Fig — (JPG) [file pone.0226619.s005.JPG]
